# Co-Infection with 4 Species of Mycobacteria Identified by Using Next-Generation Sequencing

**DOI:** 10.3201/eid2711.203458

**Published:** 2021-11

**Authors:** Lulan Wang, Dakai Liu, Lok Yung, George David Rodriguez, Nishant Prasad, Sorana Segal-Maurer, Vishnu Singh, Ellee Vikram, Angela Zou, Genhong Cheng, William Harry Rodgers

**Affiliations:** University of California, Los Angeles, California, USA (L. Wang, E. Vikram, A. Zou, G. Cheng);; New York-Presbyterian Queens Hospital, Flushing, New York, USA (D. Liu, L. Yung, G.D. Rodriguez, N. Prasad, S. Segal-Maurer, V. Singh, W.H. Rodgers);; Weil Cornell Medical College, New York (W.H. Rodgers)

**Keywords:** Tuberculosis, TB, nontuberculous mycobacterial pulmonary disease, next-generation sequencing, multiple mycobacteria co-infection, bacteria, tuberculosis and other mycobacteria, United States

## Abstract

We identified co-infection with 4 species of mycobacteria in a woman in New York, New York, USA, by using next-generation sequencing. This procedure is useful for identifying co-infections with multiple mycobacteria, tracing the geographic origin of strains, investigating transmission dynamics in susceptible populations, and gaining insight into prevention and control.

Mycobacteria are major human pathogens; ≈13 million persons in the United States live with *Mycobacterium tuberculosis* complex (MTBC) infection, and incidence of nontuberculous mycobacterial (NTM) pulmonary disease is increasing worldwide. The challenges of managing MTBC and *M. avium* complex (MAC) co-infection are well described, including the risk for falsely interpreted Xpert RIF (rifampin) results (*1*,*2*). MTBC and *M.* *abscessus* co-infection has been described in case reports only (*3*,*4*). We describe co-infection with 4 species of mycobacteria.

In July 2019, an 82-year-old Asian woman was hospitalized in Flushing, New York, USA, for persistent fever associated with worsening weakness. Computed tomography of her chest showed near-complete atelectasis of the left upper lobe, hyperinflation in other areas, and a small left-sided pleural effusion. Scattered nodular opacities in a tree-in-bud pattern and pulmonary granulomas were present throughout the lungs, and discontinuity of the left upper lobe bronchus was noted. Cultures of blood, urine, stool, and respiratory specimens yielded negative results for nonmycobacteria.

In a sputum sample collected for routine mycobacterial testing, fluorochrome staining exhibited rare acid-fast bacilli, and MTBC was detected by using Xpert MTB/RIF (Cepheid, https://www.cepheid.com). We then inoculated a Lowenstein-Jensen Gruft slant with sputum, incubated it at 37°C, and inoculated VersaTREK Myco bottles containing Modified Middlebrook 7H9 Broth with Sponges (Thermo Fisher, https://www.thermofisher.com) and incubated them at 35°C. No isolate was recovered from the Lowenstein-Jensen Gruft slant. Only MAC was detected by AccuProbe (Hologic, https://www.hologic.com) in Kinyoun-positive culture from the Myco bottles. One week later, another sputum sample with Kinyoun-positive growth from the Myco bottles was negative for MAC, MTBC, *M.* *gordonae*, and *M.* *kansasii*. *M.* *abscessus* was identified on the Lowenstein-Jensen Gruft slant.

Considering the sensitivity limit and narrow species coverage of AccuProbe and the difficulty of identifying mycobacteria by culturing and because of growth interference among different mycobacteria, we conducted next-generation sequencing (NGS) by using Hiseq3000 (Illumina, https://www.illumina.com) on the supernatant of the first sputum culture. NGS yielded ≈175 million reads, each with a quality score of >35. We checked NGS data for quality control by using FastQC (Galaxy, https://usegalaxy.org). All steps and programs used the data processing pipeline from Galaxy, an open-source, web-based platform for data-intensive biomedical research. Each read identified had a quality control score of 39.4 and an average guanine-cytosine content of 68%. Only 0.69% of bases resulted in no hits and were not identifiable. We performed De Novo classification by using De Novo Assembly Unicycler, Quast QC, and Kraken Classification (Galaxy) and generated coverage and depth data by using BWA Aligner and SAMtools Depth (Galaxy). We aligned the reads, visualized onto bacteria reference genomes by using Bowtie2 (Galaxy) and converted into BED (Browser Extensible Data) files followed by Bedtools Merge, Multicov (https://bedtools.readthedocs.io).

The genome visualization pipeline confirmed 4 genomic traces of *Mycobacterium* strains (Figure): *M. yongonense* strain 05-1390 (GenBank accession no. NC_021715.1), *M. tuberculosis* strain FDAARGOS_757 (GenBank accession no. CP054013.1), *Mycobacterium* sp. MOTT36Y (GenBank accession no. NC_017904.1), and *M. abscessus* ATCC 19977 (GenBank accession no. CU458896.1). *M. yongonense* was identified with a genome coverage of 88.73% (4.9 Mb mapped of 5.5-Mb genome) and a read depth of 1,224×. *M. tuberculosis* was identified with a genome coverage of 99.99% (4.4 Mb mapped of 4.4-Mb genome) and a read depth of 63×. *Mycobacterium* sp. was identified with a genome coverage of 94.41% (5.3 Mb mapped of 5.6-Mb genome) and a read depth of 1210×. *M.* *abscessus* was identified with a genome coverage of only 2.75% (0.14 Mb mapped of 5.1-Mb genome) and a read depth of 8× (Table). The mycobacteria identified by NGS were verified by various mycobacteria tests.

**Figure:**
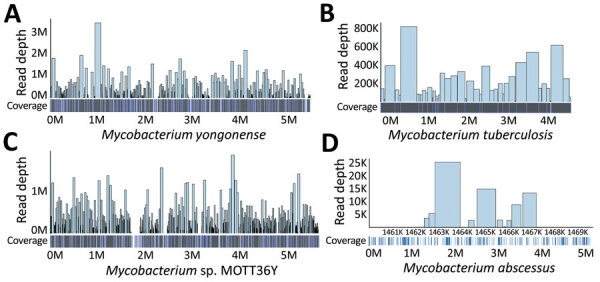
Genomic coverage and depth map of 4 *Mycobacterium* strains identified by using next-generation sequencing on isolates from a woman in New York, New York, USA. The reads were aligned by using bacteria reference genomes with Bowtie 2 and visualized by using aligned BED file (https://bedtools.readthedocs.io). A) *M. yongonense*; B) *M. tuberculosis*; C) *Mycobacterium* sp. MOTT36Y; D) *M. abscessus*.

**Table:** Classification and coverage of multiple *Mycobacterium* strains identified by using next-generation sequencing of isolates from a woman in New York, New York, USA

Strain	Genome size, bp	Coverage, bp	Coverage, %	Read depth	Quality
*Mycobacterium avium* complex sp YG	5,521,023	4,900,000	88.752	1223.91×	39.4
*M. tuberculosis* complex	4,405,981	4,405,474	99.999	63.32×	39.4
*Mycobacterium* sp. Mott36Y	5,613,626	5,300,000	94.413	1209.57×	39.4
*M. abscessus*	5,090,491	139,997	2.750	7.66×	39.4

We obtained the consensus sequence for 4 strains of bacteria by using MEGAHIT (Galaxy) and generated a BLAST (https://blast.ncbi.nlm.nih.gov/Blast.cgi) tree based on minimum evolution at the species level by using >15 kbp from each sequence. Assembly on MTBC sequencing data yielded a total consensus sequence of 4,376,826 bp and 78,208 single-nucleotide polymorphism sites (1.79%). Analysis by BLAST and Mykrobe (https://www.mykrobe.com) revealed that the MTBC isolate belongs to sublineage 2.2.

The patient received RIPE therapy (rifampin, isoniazid, pyrazinamide, and ethambutol), along with amikacin, tigecycline, and azithromycin. At 6 months, RIPE therapy was completed. At 9 months, sputum culture was negative. The patient continues to take amikacin, tigecycline, and azithromycin as an outpatient with close follow-up.

Identification of co-infection with mycobacteria is necessary for diagnosis and treatment (*5*). Treatment regimens and duration remain species specific because of unique resistance mechanisms. To achieve the greatest potential for success while minimizing toxicities, early empiric treatment should account for clinical characteristics of MTBC and NTM co-infection and strain identification.

Our report highlights the value of NGS for identifying multiple mycobacteria co-infections in populations with high susceptibility to and prevalence of MTB and NTM (i.e., immigrants, immunocompromised patients, and international travelers). NGS can trace the geographic origin of the *Mycobacterium* strain. These features, in combination with a patient’s epidemiologic exposure and travel history, could elucidate the potential time and location of infection acquisition. NGS could also be used to identify drug-resistance genes to guide targeted therapy.
